# Necrotising Fasciitis Following Sinus Laser-Assisted Closure Treatment for Pilonidal Disease: A Case Report

**DOI:** 10.7759/cureus.114407

**Published:** 2026-08-12

**Authors:** Marion Suenaert, Sandra Müller, Marie Gaillard, Sylvie Van den Broeck, Niels Komen

**Affiliations:** 1 Faculty of Medicine and Health Sciences, Antwerp University Hospital, Antwerp, BEL; 2 Department of Abdominal, Pediatric and Reconstructive Surgery, Antwerp University Hospital, Antwerp, BEL

**Keywords:** necrotising fasciitis, pilonidal disease, postoperative infection, proctology, sinus laser-assisted closure

## Abstract

Sinus Laser-Assisted Closure (SiLaC®) is a minimally invasive treatment for pilonidal disease, characterised by low reported rates of complications. This case report details a 49-year-old male who developed necrotising fasciitis following SiLaC. Within 24 hours postoperatively, the patient presented with severe pain, high fever, and cellulitis; however, crepitus was absent. Imaging revealed subcutaneous air extending up to the twelfth thoracic vertebra. Urgent surgical debridement and drainage were performed, accompanied by the initiation of broad-spectrum antibiotic therapy. Cultures subsequently identified *Streptococcus dysgalactiae*, allowing for targeted antibiotic management. The patient achieved a favourable recovery. To the best of our knowledge, this represents the first reported case of necrotising fasciitis as a complication of the SiLaC procedure.

## Introduction

Sinus Laser-Assisted Closure (SiLaC®) is a fairly new minimally invasive treatment for pilonidal sinus disease (PSD). The literature reports low recurrence and complication rates, with postoperative infections and abscesses described in only 4-5% of cases [1,2]. Despite its safety and outpatient applicability, severe complications, though rare, warrant reporting to the surgical community. We present the first reported case of necrotising fasciitis following SiLaC, highlighting the importance of vigilance in managing patients treated with this procedure. This case was previously presented at the Belgian Surgical Week 2025.

## Case presentation

**Figure 1:**
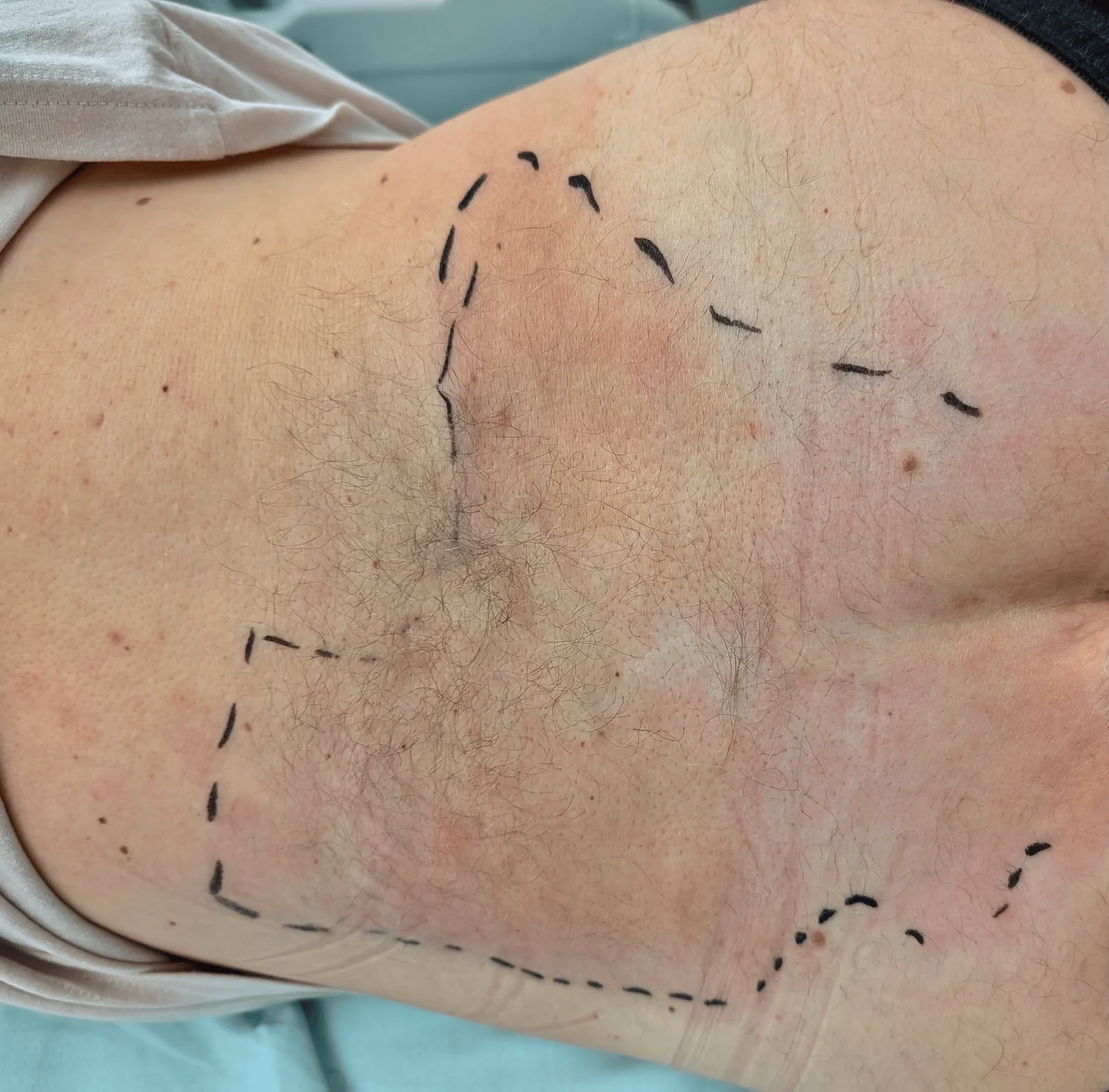
Clinical photograph of the cellulitis observed the day after admission.

A 49-year-old man with no surgical history underwent SiLaC under general anaesthesia for chronic PSD. The procedure was uneventful, utilising a 1470 nm diode laser with a total applied energy of 723 J. The patient was discharged on the same day. On postoperative day one, he presented to the emergency department with severe surgical site pain and fever (39.4°C). Blood examination revealed a white blood cell count of 9.2 × 10⁹/L and a C-reactive protein (CRP) level of 31.3 mg/L. During physical examination, cellulitis surrounding the surgical site was visible (Figure 1), without fluctuance, crepitus, or pus discharge. Broad-spectrum intravenous antibiotics (amoxicillin-clavulanic acid), fluids, and analgesics were initiated. 

The following day, the cellulitis had not progressed, and the patient’s pain had decreased. An ultrasound was ordered to rule out any fluid collections, which revealed subcutaneous air and was subsequently followed by an urgent computed tomography (CT) scan.

**Figure 2:**
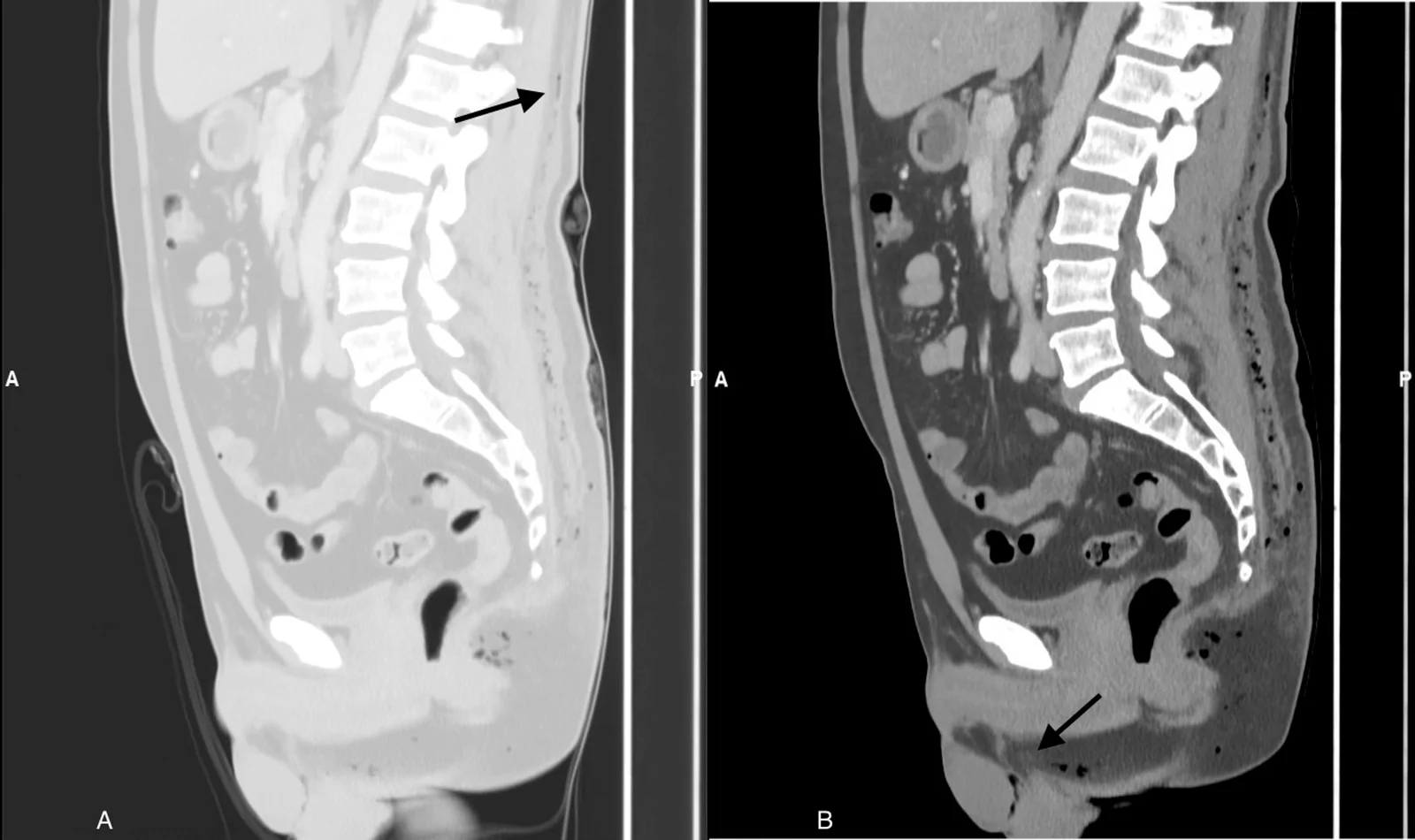
Sagittal view of multislice abdominal CT scan with intravenous contrast, showing subcutaneous air extending from the level of the coccyx up to the T12 vertebra (a), as well as at the perineum and along the right spermatic cord (b).

**Figure 3:**
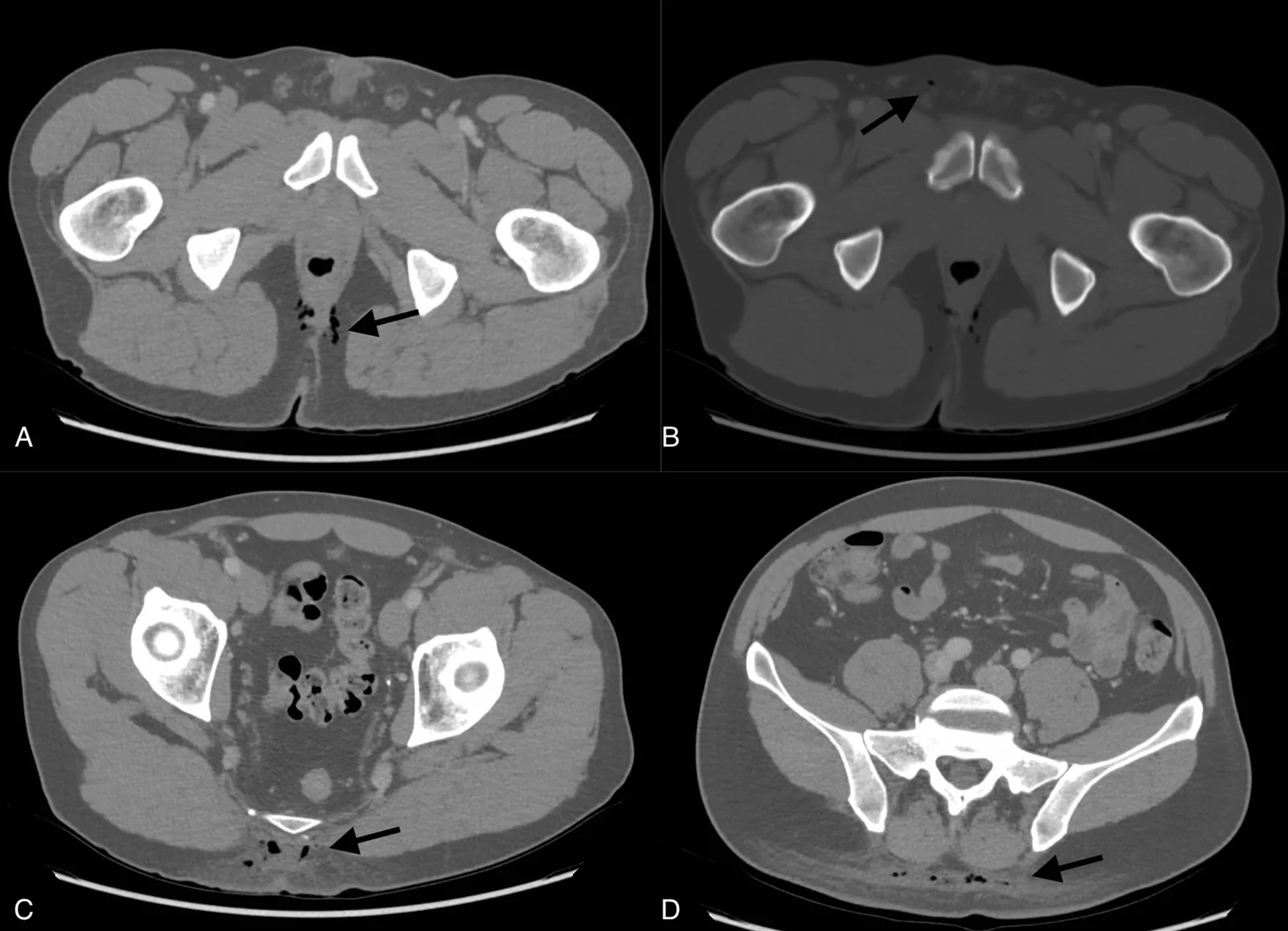
Axial view of multislice abdominal CT scan with intravenous contrast, showing subcutaneous air at the perirectum (a), right spermatic cord (b), coccyx (c), and sacrum (d).

CT imaging confirmed subcutaneous air extending from the pilonidal sinus to the twelfth thoracic vertebra (Th12) (Figure 2), as well as perirectal, perineal, and right spermatic cord involvement (Figure 3). No abscess or muscle inflammation was noted. Surgical treatment was undertaken on the same day, consisting of excision of the sinus, drainage of underlying collections, thorough debridement, and placement of a tubular drain. Tissues beyond the sinus itself were viable and required no further debridement. There was no muscle involvement. Antibiotics were escalated to piperacillin-tazobactam and clindamycin per the centre’s necrotising fasciitis guidelines [3]. *Streptococcus dysgalactiae*, sensitive to clindamycin, was identified in blood and perioperative cultures.

There was a positive clinical and biochemical evolution postoperatively. CRP levels rose to 247.9 mg/L and subsequently decreased to 36.5 mg/L at discharge. Antibiotic therapy was de-escalated to oral clindamycin five days postoperatively. The patient was discharged on postoperative day six. Wound care at discharge included twice-daily irrigation of the drainage wound with diluted Iso-Betadine® and application of Iso-Betadine® gel to the excision wound. The wound was covered with a grease-impregnated bandage.

**Figure 4:**
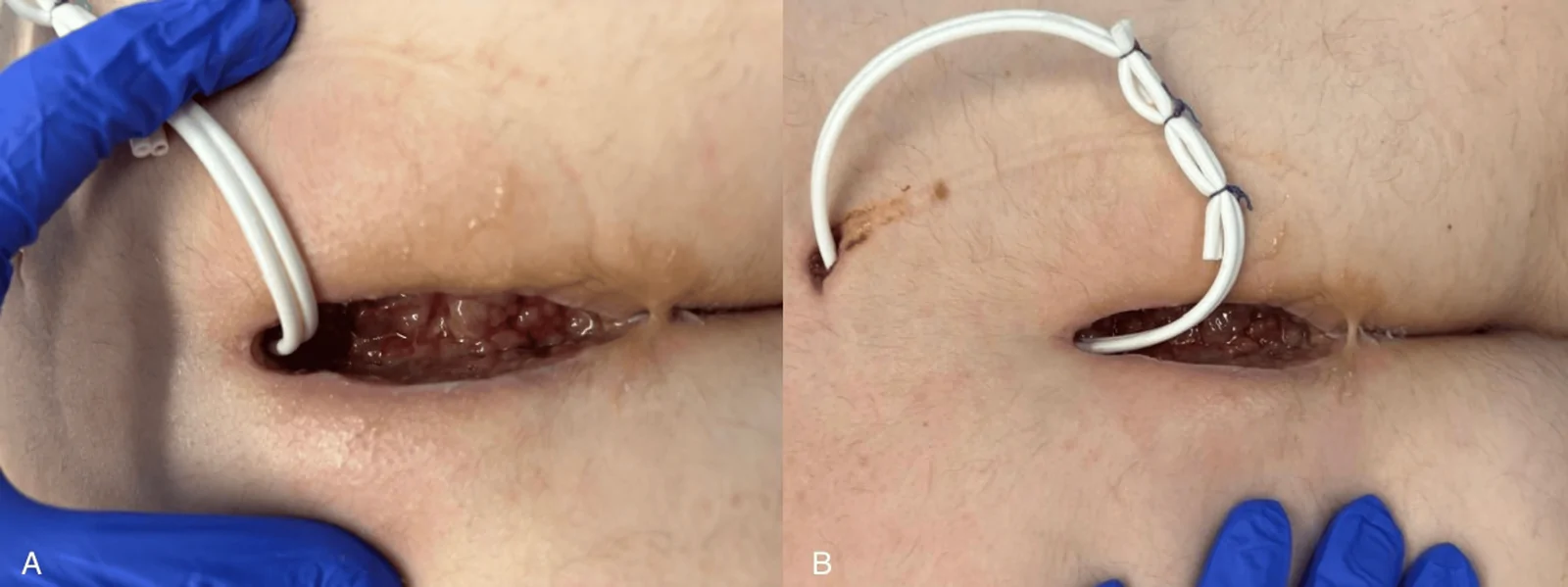
Clinical photographs of the wound as seen at the postoperative follow-up examination.

A follow-up examination two weeks later showed a well-healing wound (Figure 4). Wound care was adjusted from Iso-Betadine® gel to Flaminal Hydro®. Further wound healing was uneventful. 

## Discussion

*Streptococcus dysgalactiae* is a group G streptococcus (GGS), often found colonising the skin and gastrointestinal tract. The equisimilis subspecies is a known pathogenic organism with an infection spectrum similar to *Streptococcus pyogenes *[4]. Though among the most common disease manifestations are non-invasive skin and soft tissue infections, cases of necrotising fasciitis have been reported, and a relative increase in the proportion of bacteraemia due to GGS has been shown in a retrospective study [5,6]. A review of the available literature revealed no other cases of necrotising fasciitis following SiLaC specifically, but it has been reported with similar procedures [7,8]. It can be hypothesised that, in this case, *Streptococcus dysgalactiae *was present in the sinus prior to the SiLaC procedure. As it was sealed inside due to the thermal effect of the laser, rapid bacterial growth with release of endo- and exotoxins followed, resulting in the presented clinical and radiological findings.

## Conclusions

This case illustrates the rare but severe complication of necrotising fasciitis following SiLaC for PSD. Early recognition, prompt surgical intervention, and appropriate antibiotic therapy were crucial in ensuring a favourable outcome. Despite its low complication rate, treating physicians should remain vigilant for severe complications. This case report highlights the importance of awareness to ensure timely identification and management of such risks.
